# Impacts of Cognitive Factors on Creativity Quality in Design: Identification from Performances in Recall, Association and Combination

**DOI:** 10.3390/jintelligence11020039

**Published:** 2023-02-20

**Authors:** Yuan Yin, Haoyu Zuo, Peter Childs

**Affiliations:** Dyson School of Design Engineering, Imperial College London, London SW7 2AZ, UK

**Keywords:** creativity, creative design, design cognition

## Abstract

The reason why people have different creativity quality levels may depend on their different performances relating to other cognitive factors that are important for creativity. This study was designed to identify the performance of three cognitive factors (recall, association, and combination) that a designer may use in a creative process and then identify how the differing performance for these cognitive factors will affect creativity quality levels. Seventy-one participants were recruited to undertake a design task and complete a semi-structured interview. The results indicate that, in a creative design process, similar performances in recall, association, and combination can result in differences in creativity quality level.

## 1. Introduction

Creativity can be regarded as the ability to imagine or invent something novel and valuable (Sarkar and Chakrabarti 2008; Jung et al. 2010). Understanding how creativity occurs can help people to generate more ideas (Childs et al. 2022). Creativity is related to human psychological and cognitive processes (Finke et al. 1992; Gaut 2010; Casakin and Kreitler 2011; Perlovsky and Levine 2012); it is a dynamic process that is partially out of conscious control (Dietrich 2004; Mok 2014; Brem and Puente-Díaz 2020).

Some cognitive process models of creativity have been summarized, such as four-stage models (Wallas 1926; Basadur and Gelade 2005; Miller 2014), dual-process models (Guilford 1956; Campbell 1960; Basadur et al. 1982; Finke et al. 1992; Howard-Jones 2002; Gabora 2010; Nijstad et al. 2010; Gabora et al. 2014), tripartite-process models (Leschziner and Brett 2019), and cognitive factor process models (Bhattacharya and Petsche 2005). Other studies have focused on which cognitive factors are involved in the creativity process. Cognitive style (Glass and Riding 1999; McKay et al. 2003; Sagiv et al. 2010; Gilhooly 2016), attention (Carson et al. 2003; White and Shah 2006; Vartanian et al. 2007; Gabora 2010; Moraru et al. 2016; Carruthers et al. 2018), short-term memory (Gubbels et al. 2017; Mao et al. 2020), working memory (Takeuchi et al. 2011; De Dreu et al. 2012), semantic memory (Beaty et al. 2020), episodic memory (Fink et al. 2009; Madore et al. 2015; Benedek and Fink 2019), association (Guilford 1956; Finke et al. 1992; Nijstad et al. 2010; Liu 2016; Benedek et al. 2020), combination (Wan and Chiu 2002), and cognitive load (Hirschi and Frey 2002; Redifer et al. 2021) have been identified as the cognitive components of the creativity process.

However, although some designers follow the same cognitive processes (such as first undertaking divergent and then convergent thinking) in a creative process, the creativity quality levels of designers’ outputs are still different. Additionally, some designers exhibit the same cognitive factor (such as remote association) processes to generate creative ideas, while the creativity quality levels of designers’ outputs are also different. In other words, existing research cannot explain why people following the same cognitive processes or relying on the same cognitive factors to generate creative ideas have differing creativity output quality levels.

Current cognitive process models do not give a systematic explanation for what happens during a specific cognitive factor process (such as the association process, combination process, or recall process). Therefore, this study aims to identify the performances of cognitive factors that a designer may have in a creative design process. Furthermore, the study explores how differing performances of cognitive factors will affect creativity quality levels. The results of this study will contribute to helping researchers and designers understand which kind of performance is more likely to be related to higher creativity output quality levels.

## 2. Literature Review

To understand creativity, attempts have been made to explain the occurrence of a creative process in terms of cognition (Miller 2014). In this section, cognitive process models of creativity are reviewed. The creative process is a complex cognitive process (Dinar et al. 2015). Often, creative ideas are generated based on a combination of divergent and convergent thinking processes. The divergent thinking process is the core creative idea-generation process, which is about finding different creative solutions to problems (Said-Metwaly et al. 2022). The convergent thinking process is mainly about the selection and refinement of solutions (Benedek and Fink 2019).

### 2.1. Blind Variation and Selective Retention Model (BVSR)

The blind variation and selective retention model (BVSR) involves “generating ideas blind (blind variation)” and “selecting the fittest variants to develop a final product (selective retention)” processes (Simonton 2010). Blind variation processes suggest that creative tasks will not affect the idea-generation process. Ideas will be generated based on memory instead of a combination of memory and creative tasks. No matter which creative tasks were set, the ideas generated initially were the same. Then, selective retention processes can retain new and hopeful ideas. Based on this model, increasing the number of initial ideas can be helpful for designers to select more useful ideas (Nijstad et al. 2010).

### 2.2. Genoplore Model

The Genoplore model includes generation and exploration processes (Finke et al. 1992). The generation process in the Genoplore model is a divergent thinking process that involves searching long-term memory, forming associations, synthesis, and transformation. The exploration process is a convergent thinking process where potential functions of ideas are considered. This model indicates that people tend to rely on examples in the same or similar areas as inspirations for new ideas (Ward and Kolomyts 2010). This inspiration process is an association process, which indicates the importance of association ability in creativity (Rossmann and Fink 2010; Benedek et al. 2017).

### 2.3. Gabora’s Model

Gabora’s model (Gabora 2010) pointed out that, in idea-generation processes, people autonomously associate highly and remotely relative items from memory based on stimulation (flat association), select ideas based on individual characteristics and current conception, refine the selected idea, and connect it with task demands through associative and analytic thinking (Gabora 2005). Gabora’s model is often compared with the BVSR. Both of the models suggest that idea-generation processes often include a refinement process; however, the idea-generation processes are different between the two models. This difference is located in the relationships between initial ideas and creative tasks. The BVSR proposes that initial ideas are generated without context, while the initial ideas of Gabora’s model are developed based on creative tasks.

### 2.4. Structure of Intellect Model

According to the structure of intellect model (Guilford 1956), creative processes include divergent and convergent thinking processes. Divergent thinking processes are an associative process. In these processes, the encoded information is combined with information in the current context and the attention is distracted (Gabora 2010). Convergent thinking processes involve analyzing and are important in detailing and evaluating ideas. In this model, Guilford supports the view that divergent thinking processes occur before convergent thinking processes.

### 2.5. Cognitive Factor Process Model

Bhattacharya and Petsche (2005) divided the cognitive process of creativity into three sub processes: “Rely on long-term memory to remember more patterns”; “Rely on visual memory or short-term memory to maintain”; and “Generate possible graphic and active visual memory”. This cognitive model indicated that people tend to first have an idea in mind and then draw it (Gombrich [1960] 2000); however, this model cannot explain why the final idea may differ from the initially imagined idea. This may be explained by the existence of imagined idea–expression processes, where imagined ideas are improved. Another limitation of this model is that the cognitive process involves few cognitive factors, such as attention, novel combinations, and aesthetic evaluations (Damasio 2001); however, this model only explains how memory is involved in a cognitive process of creativity and ignores the effects of other cognitive factors.

### 2.6. Nijstad et al.’s Model

Nijstad et al.’s model reported that cognitive processes of creativity are constituted of flexibility and persistence pathways. Flexibility pathways help people generate more categories of ideas through remote association (Nijstad et al. 2010). This flexibility pathway can partly explain why people can generate various ideas and have variants. In persistence pathways, systematic thinking and incremental searching are used to evaluate creativity. The ideas that can be obtained from systematic thinking and incremental searching are eliminated. The remaining ideas are those which have the potential to be creative ideas. People may not collect enough potential creative ideas initially. To obtain more potential creative ideas, people may return to the flexibility pathway and cycle the two pathways until the ideas are sufficient. In cycling, a high shifting ability between flexibility and persistence pathways is required. Flexibility pathways require higher focused attention than persistence pathways. Additionally, people can make full use of creative-task-related information in flexibility pathways. In persistence pathways people need to exclude information that is not related to a creative task and select the most suitable idea.

### 2.7. Research Gaps

From a review of cognitive process models in creativity, it can be identified that memory (especially long-term memory (LTM)), association, and combination are the three main cognitive factors that are likely to be included in cognitive process models in creativity (Table 1).

LTM is memory that has been stored in the brain for a long time (Norris 2017). Memory is one of the fundamental elements of creativity (Beaty et al. 2017; Benedek and Fink 2019). Although simply recalling previous memories does not lead to creativity, creativity does not come ex nihilo. People rely on their previous knowledge to create new things (Gabora 2005). To simplify expression, in this paper recall is used to represent memory, especially LTM. Association is an important cognitive factor in creative processes (Guilford 1956; Mednick 1962; Finke et al. 1992; Nijstad et al. 2010; Liu 2016). Association comes in the forms of remote and common associations (Benedek et al. 2020). Remote association is the ability to associate not-related concepts, while common association is the ability to associate related concepts. Most people can associate highly related items from memory based on stimulation, while only creative people may associate remotely related items from memory based on stimulation (Gabora et al. 2014). Combination is a cognitive process where two or more concepts are mentally synthesized into a new concept. Designers with novel combination ability are more likely to have higher creativity quality levels (Wan and Chiu 2002).

Although three cognitive factors (recall, association, and combination), which are mainly related to the cognitive process of creativity, have been found, existing research still has some limitations. The findings among cognitive process models are inconsistent (Abraham 2022), and they cannot explain why people following the same cognitive processes may display different creativity quality levels. This may be because the cognitive factors that are components of cognitive processes are not fully identified. Thus, some researchers have called for an understanding of the cognitive process of creativity through the further understanding of cognitive factors (Abraham 2022). Therefore, it is worth further exploring the cognitive process in creativity by understanding the performance of cognitive factors.

### 2.8. Research Aims

This study aims to identify the detailed performance in three cognitive factors (recall, association, and combination) in a creativity design process and its relation to creativity quality levels. To address the research aims, participants were asked to finish a design task; they then needed to conduct a semi-structured interview in order to detect the performance of recall, association, and combination in a creative process. The performance aspect that this study focused on was mainly the contextual details of cognitive factors. The term “contextual details” refers to what participants have thought during the specific cognitive factor process.

## 3. Methods

To achieve the research aims, 71 participants were recruited to finish a design task. A semi-structured interview was subsequently conducted in order to identify their cognitive factor performance in the design process.

### 3.1. Participants

Seventy-one (23 male, 48 female; aged 20–29) design-background Chinese students were recruited in this study. All of the participants had finished more than one creative design task and used hand drawing methods (such as sketching) to express their ideas within the preceding year.

### 3.2. Methodology

To achieve the research aims, participants first needed to undertake a design task and then a semi-structured interview. This section introduces the design task and the semi-structured interview.

#### 3.2.1. Design Task

In the design task, participants were asked to “design a product using the provocation of the word ‘fish’ within one hour”. This task was one of China’s 2019 design major graduate student admission exam tasks. All of the participants reported that they had not conducted this design task before.

#### 3.2.2. Semi-Structured Interview

To identify the performances of cognitive factors, existing studies have mainly been based on measurements that can reflect the performance in real time (Pringle and Sowden 2017). Thinking aloud is one of the representative methods for this measurement, as it can report the cognitive processes during a flow of activity (Koro-Ljungberg et al. 2013); however, asking participants to self-report their cognitive processes may increase the cognitive load on participants and thus affect creative design processes. It may not be possible to identify why some cognitive factors occur from a think-aloud process (Young 2005). These may be reasons why existing research has not reported the performances of cognitive factors in-depth and one of the reasons for the research gaps mentioned. Therefore, this study tried to apply some other research methods to understand the performance of cognitive factors during a creative design process.

Post-experiment interviews are often used to collect data. This offers a chance to directly elicit participants’ cognitive thinking processes, such as opinions, thoughts, and feelings, behind cognitive processes in-depth (Easwaramoorthy and Zarinpoush 2006). For this reason, a semi-structured interview was conducted following the completion of the design task; however, interviews cannot reflect participants’ real-time conditions, and participants may have the tendency to give what they perceive to be desirable responses. To mitigate these limitations, the study asked participants to answer the questions based on their real conditions instead of perceptions. Additionally, to avoid participants not remembering what they have thought during the design processes, the creative design processes of participants were recorded. Participants were permitted to review the creative design processes as a prompt if they thought that they could not remember what had happened.

In the interviews, participants were asked to provide information on three cognitive factors (recall, association, and combination). Participants were first introduced to what recall, association, and combination were in order to ensure that they understood what the focus of the interview was. As some of the terms may have been hard for participants to understand, some simplified explanations were used. To be specific, “recall” was explained as when participants searched for some information, graphics, sentences, experience, events, or scenes from their memory; “association” was explained as when participants try to associate irrelative (remote association) or relative concepts (common association); and “combination” was explained as when participants try to combine some information, graphics, or sentences to generate a concept that is incompatible and empty in life (remote combination) or not incompatible in life (common combination).

Questions were then asked to establish an in-depth understanding of cognitive factors (recall, association, and combination). For each of the three cognitive factors investigated in this paper (recall, association, and combination), we wanted participants to tell us how they knew they needed that factor, what it consisted of, what the results of it were, which words they would use to describe it, and what the difficulties were that they encountered in dealing with it.

**Figure 1:**
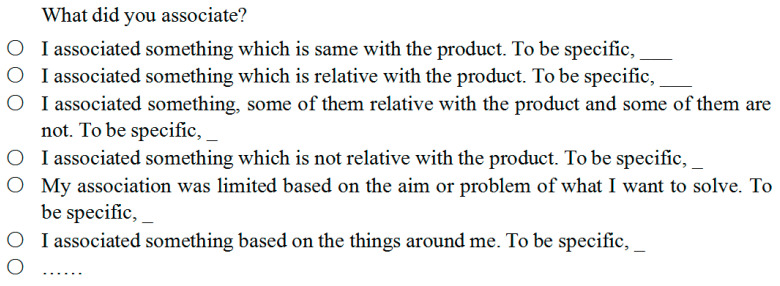
An example of a question and its semi-open answers (the study was conducted in Chinese, and the example is a translated version).

“How to make participants understand the questions” was a challenge. Providing multiple answers to each question may help participants understand the interview questions; however, this solution introduced new problems. Participants may rely on the given options to answer and be slothful to recall or think about what they actually did. In addition, participants sometimes were limited to the given results (Köhnken et al. 1995). Their answers may not reflect their actual experiences. Therefore, to reduce the limitation of this solution, the provided answers were not full; instead, they were semi-open answers. Participants were asked to complete some parts of their answers based on their experiences in the creative design process. One example question and its semi-open answers are shown in Figure 1.

### 3.3. Procedure

After reading the information sheet, which introduced the research project and its aims, in addition to clearly outlining the entire research process, participants could ask questions for clarification. If there were no questions they were asked to sign the consent form. A link to the online questionnaire link was then sent via email. The questionnaire was deployed using Qualtrics.

The first part of the questionnaire was designed to collect the participants’ basic information, including their educational background and creativity design experience. It took approximately 5 min to finish this part of the questionnaire. Participants who had experience in conducting a creative design task in the preceding year and had experience in expressing their ideas using hand drawing methods (such as sketching) were selected as participants.

The participants were then asked to gather all of the materials needed for the design task and to complete the second part of the questionnaire, which is the material report. Next, they were asked to design a product using the provocation of the word “fish” within one hour and draw the product as a paper-based sketch. Participants were informed that there were no limitations on which kind of product they needed to design as long as they thought their design could fulfill the design task. Participants were also informed that they could draw their ideas in color or black and white. Additionally, they could use any drawing tools as long as they were types of paper sketching tools. They were also told that the drawing quality and form will not affect the assessment of their creative outputs’ creativity quality levels. In addition to the final idea, participants were also urged to draw any ideas that flashed through their minds, as well as any other ideas that they thought of during the design process, using simple forms or a few words as appropriate. They were informed that the final idea would not be used for creativity evaluation.

After the design task was complete, each participant underwent a semi-structured interview. Participants were first introduced to the meaning of recall, association, and combination. They were then asked to answer the interview questions. Finally, participants self-evaluated their results and whether they expressed their ideas clearly.

All participation was voluntary in this study. The interview was recorded via online platforms that can provide voice and video communication, such as Zoom, WeChat, QQ, Teams, or Tencent Meeting. For each participant the study lasted around one and a half hours. With participant permission, the whole process was recorded.

### 3.4. Creativity Output Assessment

#### 3.4.1. Participants

After all of the participants’ creative outputs were collected, five experts (three females, two males, aged 31–40), who had worked in product design areas for more than 10 years and had experience in assessing over 50 creative design outputs in the last year, were recruited to assess the 71 creative outputs. “Creative outputs” refer to the outputs generated from the creative design process.

Experts were considered to be raters because they can assess creative work efficiently and effectively (Amabile 2018; Han et al. 2019; Kaufman et al. 2008; Runco 1994). A decision on the number of raters needed to be made. Some research studies have used two expert raters (Charyton and Merrill 2009), four experts (Doré et al. 2007), five experts (Ahsan et al. 2020; Han et al. 2019; Sarkar and Chakrabarti 2011), or more (Baer et al. 2004). As there is no standard for how many raters are required to assess creativity (Lai et al. 2006), this study recruited five experts in an attempt to adequately conduct the assessment.

Similarly, there is no standard for the relationship between raters’ experience and whether they are considered an expert. Some research has mentioned that they recruited experts who had four years (Sarkar and Chakrabarti 2011), five years (Han et al. 2019), or ten years of experience (Han et al. 2019) in a relevant creativity area as raters. This study selected raters who had worked in product design areas with more than 10 years of experience in order to ensure that they were able to assess creativity at a more advanced professional level.

#### 3.4.2. Assessment Criteria

The creative product semantic scale (CPSS) method (Besemer and O’Quin 1987), which is a seven-point Likert scale, was selected to assess the participants’ creative outputs. The original CPSS included 3 catalogues, 11 subscales, and 55 items. Chulvi et al. (2012) selected 18 items related to novelty and usefulness as the abbreviated CPSS criteria for assessing product design outputs (Han 2018; Yin et al. 2021; Table 2).

#### 3.4.3. Protocol

Each expert first received the information sheet and consent form. They could ask any questions for clarification. If there were no questions, or once they had had any questions satisfactorily answered, they signed the consent form. The assessment of creative outputs was conducted based on a Qualtrics online questionnaire platform. The first part of the questionnaire collected the experts’ basic information.

In the second part, five experts were asked to assess all 71 creative outputs. This section first introduced how the 71 creative outputs were collected—all of the creative outputs were generated from the design task, which was to design a product inspired by the topic “Fish” within one hour. Experts were then instructed as to how to assess the creativity outputs by using the CPSS—assessing the product by finishing a list based on a bipolar seven-point scale. The scale is shown in Table 2. There were no time limitations for completing each design output CPSS assessment.

The 71 assessment tasks were included in one questionnaire; however, the participants were informed that they did not need to assess all 71 creative outputs at once. Instead, they were given a week to finish all of their assessments. They were advised that they could pause the assessment at any time and return to it later using the same link. All of the participants finished the assessments in a week.

## 4. Results

This section provides the results of the creative output assessment and the interview, and includes three subsections: the design output assessment results, the creativity quality level results, and the interview results.

### 4.1. Output Assessment Results

The descriptive statistics included minimum, maximum, mean, and standard deviation (SD) values (Table 3).

Some experts were severe in their judgments and associated scoring, while others were lenient. It was therefore necessary to normalize the original assessment results, such that these data could be aligned to identify the probability distribution. The min-max normalization method (y = (x − min)/(max − min)) was used to accomplish this aim, as it is one of the most common normalization methods and suited to this task. SPSS was used for the calculation (Table 3). Cronbach’s alpha is a statistical method that is used to assess the degree of internal consistency among results (inter-rater reliability; Rosaroso 2015). In other words, Cronbach’s alpha is used to present to what level one participant’s results can predict the remaining participants’ results. The higher the Cronbach’s alpha, the higher the internal consistency of the results. The Cronbach’s alpha of the normalized CPSS results was 0.679 (95% CI = 0.544–0.784), which indicated a moderate internal consistency of the results.

### 4.2. Creativity Quality Levels

**Figure 2:**
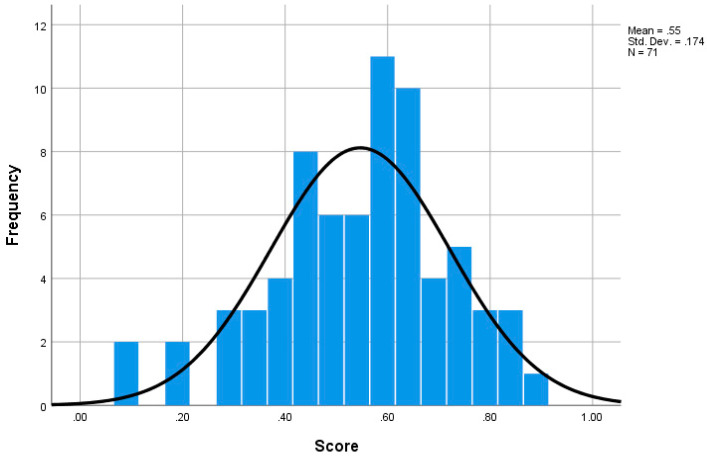
The histogram of frequency counts. The mean score is 0.55 (SD = 0.174). Cronbach’s alpha is 0.827.

The creativity score for each participant’s output was calculated from the normalized data. The mean value of the five experts’ normalized CPSS results on a particular design output is the creativity score of this output. The histogram of frequency counts is shown in Figure 2. The Cronbach’s alpha is 0.827, which indicates a very good inter-rater reliability.

The creativity quality levels are divided into five levels initially. The creativity score boundary of each level was based on the normal distribution; to be specific, they are μ + 1.5σ, μ + 0.5σ, μ, and μ − 0.5σ (Echauz and Vachtsevanos 1995).

There were two possible ways to apply the division boundary in this study—linking it to the number of participants or to the infinity samples. If the division boundary was linked to the number of participants, the boundary of each level may change as more samples are included. This makes the division unreliable. If the division boundary was linked to the infinity samples, the boundary of each level was fixed. Therefore, the paper divided the creativity quality levels based on the infinity samples. The division index is shown in Table 4.

**Figure 3:**
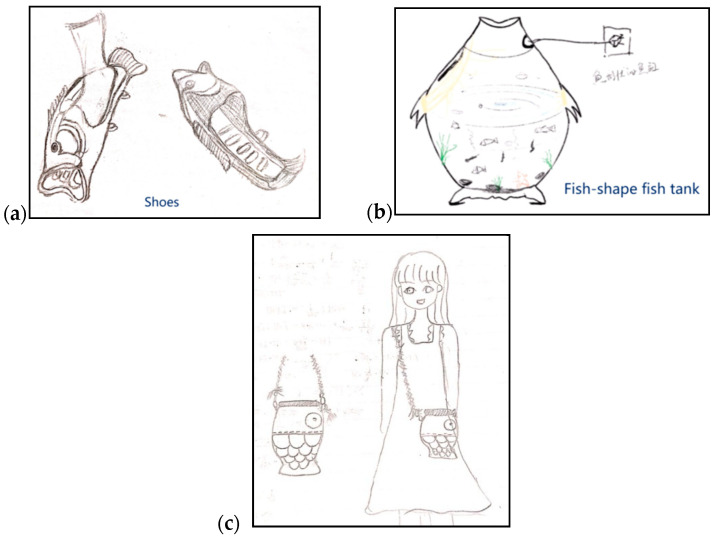
Examples of High, Middle, and Low creativity quality level results. (**a**) An example of a High level of creativity quality. (**b**) An example of a Middle level of creativity quality. (**c**) An example of a Low level of creativity quality.

None of the samples were scored as “Outstanding”, and only seven samples were scored as “Not so good”. The two levels, thus, may not have enough samples to be analyzed. Therefore, this study combined the “Outstanding” level and the “Excellent” level. The two combined levels were renamed as the “High” creativity quality level. The “Very good” level had 29 samples and was considered as the “Middle” creativity quality level. The “Satisfactory” level and the “Not so good” level were combined. The two combined levels were renamed as the “Low” creativity quality level. A chi-square test based on cross-tab can be used to detect whether the categories and results were significantly related (Bi et al. 2021). This is what this study expected; the categories in this study were the three groups and the results were scores. By using a chi-square test based on cross-tab, this study thus can identify whether the three creativity quality levels and scores are related. The chi-square test result of this division is χ2 = 142, *p* < .001, which indicates that the three creativity quality levels and scores are related. Examples of High, Middle, and Low creativity quality level results are shown in Figure 3.

### 4.3. Interview Results

The purpose of the interviews was to develop a deeper understanding of the performance of cognitive factors in creative design. To achieve this, thematic analyses were used to analyze the results from the interview after all of the interviews were conducted. The analysis processes were adopted from Braun and Clarke (2006). Researchers first familiarized themselves with data from the interviews. The three initial themes, “recall”, “association”, and “combination”, were generated. These were the cognitive factors that the study focused on. Codes were then generated based on the questions asked. How the interview questions were transformed into codes is shown in Table 5. Ten codes were assigned for “recall”; eight codes for “association”; and nine codes for “combination” (Table 5). The statements of participants which were related to the themes and codes were then extracted. NVivo software was used to assist the thematic coding process. The results are detailed and further discussed in Section 5.1.

## 5. Discussion

This study used interviews to develop an understanding of the performance of cognitive factors (recall, association, and combination) in design creativity and identify how they are linked to the levels of creativity quality. In this section, the interview results, the limitations of the study, and future research are discussed.

### 5.1. Results Discussion

In this subsection, the results from the interviews are discussed. Analyses were undertaken to explore when cognitive factors happened, how the participants realized that their cognitive factors happened, the information that the participants searched or generated in a specific cognitive factor process, the confidence levels of the results, the level of consistency of expectations and the results, and how the participants realized that they needed the cognitive factor process.

In this section, the following three short-term notations are used: “High-creativity-quality people” is used to denote the expression of participants whose design outputs were scored as being of a High level of creativity quality; “Middle-creativity-quality people” is used to denote the expression of participants whose design outputs were scored as being of a Middle level of creativity quality; and “Low-creativity-quality people” is used to denote the expression of the participants whose design outputs were scored as being of a Low level of creativity quality.

#### 5.1.1. Performance of Recall

Recall in this study is used to represent memory that has been stored in the brain. When participants searched for some information, graphics, sentences, experience, events, or scenes from their memory, a recall process happened. Seventy-one participants reported that they had recall processes during the creative design task.

**When recall happened.** Recall often happened at the beginning of a design process. Few participants mentioned that their recall processes happened when they were drawing. Twelve High-creativity-quality people mentioned that their recall processes occurred during or after their association processes (95% CI = 0.0978~0.2742). This may indicate that High-creativity-quality people recall events during or after association and use these results as a source with which to generate their ideas, while Middle- and Low-creativity-quality people do not have recall processes during or after association, and mainly rely on association results to generate ideas. Disturbances may affect the recall process. For example, participants may first recall a fish and a product. For example, during the design process of P24 the participant suddenly thought of rain, which is a “disturbance”. This disturbance triggers the memory of P24 on forgetting an umbrella on a rainy day.

**How participants realized that they recalled something.** This question was answered by participants in the interviews through the question of “How did you know you need the recall process and how did you realize you have recalled something?”. Of the 71 participants in the study, 49 reported how they realized their recall processes. Twenty-seven participants stated that they realized the recall process because they noticed that some results of the recall process appeared in their minds (95% CI = 0.2761~0.4967). Fifteen participants mentioned that they realized they recalled because they knew recall was considered to be a necessary process for design (95% CI = 0.1313~0.3209). In other words, they used the process consciously. This supports the idea that cognitive processes can be learned and applied in design. Additionally, this supports the idea that recall is a necessary process in design. The remaining participants mentioned that they realized this process because they use information generated in the recall process when they draw. Thus, they realized the existence of the recall process.

**Which kind of information was recalled by participants.** This question was answered by participants in the interviews through the question of “Can you provide more information on what you have recalled during this recall process?”. The participants’ recollections can be divided into three main categories: memories relating to the design topic, memories relating to products, and memories relating to other information. Most of the participants reported that their memories took the form of colorful graphics and text in their minds.

The design topic assigned in this study concerned fish. Therefore, in this study, memories about the design topic are memories of fish. High-creativity-quality people recall various aspects of fish, such as the characteristics of fish, their conditions of usage, fish products they have encountered before, and cartoon fish (95% CI = 0.5881~0.9724). Middle-creativity-quality people were more likely to recall a specific aspect of fish (95% CI = 0.8137~0.999). This specific aspect included a type of fish, the characteristics of fish, their conditions of usage, fish products they have encountered before, or cartoon fish. Compared with Middle-creativity-quality people, who were more likely to deeply recall information on a specific fish-related direction, High-creativity-quality people were more likely to recall broad fish-related topics. Low-creativity-quality people were more likely to recall the characteristics of fish and instant idea-related information (95% CI = 0.7627~0.9910). For example, if they decided to design a product related to a shark, they may directly focus on information about sharks. These results indicate that recalling a broad range of information has a positive effect on higher creativity quality levels.

As for memories related to the product, High-creativity-quality people were more likely to recall products that they thought may be suitable for the design topic (95% CI = 0.5168~0.9316). Middle-creativity-quality people were more likely to recall a specific kind of product (95% CI = 0.5168~0.9316), which was set by participants as their final product catalogue. Low-creativity-quality people were more likely to recall some topic-related products (95% CI = 0.6882~0.9512). The differences among High-, Middle- and Low-creativity-quality people were that High- and Middle-creativity-quality people recalled the whole shapes or functions of products and did not limit themselves to recalling products related to fish (the design topic). Low-creativity-quality people mainly recall the whole shapes or functions of fish-related products (or design-topic-related products). The differences between High- and Middle-creativity-quality people can be described as “how many kinds of products they recall”.

Concerning memories of other information (i.e., not related to fish), fewer people (11) mentioned that they recalled such memories. This may be because the design task was too easy or bounded, as well as the fact that participants had little difficulty in developing ideas based on their memories of fish or products. Therefore, they did not need to seek ideas from memories about other information. Future research on this subject should incorporate different design tasks. Among the people who reported such memories, most of them (seven) were High-creativity-quality people. The recall results of Middle- and Low-creativity-quality people were related more specifically to fish and products.

**Whether the results of recall were evaluated.** This question was answered by participants in the interviews through the question of “Do you evaluate the results you have searched in recall processes?”. Sixteen participants mentioned that they evaluated their recall results. Most of these people (13) were High- and Middle-creativity-quality people. They mainly evaluated whether the recall processes were reasonable, whether the recall directions were suitable, and whether the results were effective. When they thought their results were effective or they felt that they could not be bothered to evaluate the results further, they moved to another cognitive factor process. Five participants stated that they did not evaluate their recall results because they thought that they needed to collect as much information as they could during their recall processes.

**Participants’ confidence levels for recall results before recalling.** This question was answered by participants in the interview through the question of “How much confidence do you have in the recall results before having recall processes?”. Forty-five participants indicated that they were uncertain about what they would recall before recalling. They did not know whether their recall results would help them generate ideas because they thought the mind could not be controlled and that what they thought in recall processes may change subconsciously. Some of them (nine) explained that they were not familiar with this design task and thus could not predict their results because they could not anticipate the ideas that they would come up with. Twenty-nine participants were confident about what they would recall. For these people, their aim of recall was not to achieve detailed results but to confirm the kind of object that they wanted to design.

**Consistency between recall expectation and results.** Forty-eight participants reported that their recall results were consistent with their expectations. Seven participants reported that their recall results were not. Of these seven participants, five were High-creativity-quality people. This may be because, in the recall process, High-creativity-quality people divided their attention and were thus more likely to recall random memories that were not related to what they planned to recall initially.

**How participants realized they needed to recall.** There are three potential ways for participants to realize that they need to recall: the knowledge of design processes guided them to recall; the experience guided them to recall; and the recall process happened unconsciously. Most of the participants (42) reported that their experience guided what they would recall. This is a precondition to support the idea that when participants realize which kind of cognitive process may lead to a higher creativity quality level they will learn it and apply it to their design processes.

#### 5.1.2. Performance of Association

Association can be divided into remote association and common association. Remote association is the ability to associate usually unrelated concepts, while common association is the ability to associate related concepts (Stevens and Zabelina 2020). Fifty-nine participants reported that they had association processes during the creative design task.

**When association happened.** The data indicated that High-creativity-quality people experienced association during or after their recall processes (12 out of 14). This finding is consistent with another finding in this study that indicated that the association process is based on what an individual recalls (P36, P4, P69, and P59). Middle- and Low-creativity-quality people’s association processes may happen in various processes, such as during or after reading the design task (eight), during or after recall (31), during or after combination (six), during drawing (five), or throughout the entire process (three).

Among Low-creativity-quality people, only five of them reported that they had an association process. The remaining Low-creativity-quality people mainly combined what they recalled to generate a new product, because they knew what they wanted to design (95% CI = 0.5767~0.9077). To be specific, in this design task what they wanted was to combine, for example, was a beautiful fish appearance and a product shape. This is consistent with the results of the recall process. Most Low-creativity-quality people recalled a particular fish-related (design-topic-related) product and the associated characteristics of fish (the design topic). Therefore, compared to High- and Middle-creativity-quality people, who relied on association processes to generate ideas, Low-creativity-quality people had a relatively clear design output plan. The association process thus did not have a huge effect on Low-creativity-quality people.

**Association forms.** The aim of the association process was consistent across all of the groups, which is generating related ideas and memories. For example, P14 mentioned that “from a fish pattern and clothes, I associated where this pattern can be used, such as children’s clothes, adults’ clothes, and scenes of adults or children wearing [such clothes]”. The two kinds of associations were referred to by a similar number of participants (association based on one item: 34; associations based on a few items: 33). High-creativity-quality people (10/14) and a few Middle-creativity-quality people (11/29) are more likely to report that their associations were based on a few items. Most Middle-creativity-quality people and Low-creativity-quality people (13/28) are more likely to report that their associations were based on one item.

**What the participants associated.** High-creativity-quality people reported that their association results comprised fish-related (topic-related) information (95% CI = 0.5881~0.9724), such as shapes similar to fish, where fish can be used, the meaning of fish, products related to fish (such as boats), places where fish live (such as oceans), and fish-related lectures, presentations, or films. For High-creativity-quality people, what they tried to do was associate based on the restructured recall results. This is a kind of divergent thinking (P65).

Middle-creativity-quality people’s association results mainly concerned different kinds of fish and fish-related biology (such as birds, flowers, water plants, and shells) (95% CI = 0.5409~0.8550). Some of the associations reported by Middle-creativity-quality people were also reported by High-creativity-quality people, including places where fish live (such as oceans and lakes), the shapes of fish, and fish-related products (such as cookies). For Middle-creativity-quality people, what they wanted to do was think divergently and generate more ideas based on one item (P37).

The difference between High- and Middle-creativity-quality people is their goals of the association process. Both High-and Middle-creativity-quality people relied on common associations to generate ideas; however, for High-creativity-quality people, what they did in their mind was a remote association process, while the results were common associations. For Middle-creativity-quality people, what they did in their mind was a common association process, and the results were common associations. Therefore, although the association results for High-and Middle-creativity-quality people were both common associations, the creativity levels of common associations may be different because of the different association behaviors in the mind. This may explain why, in general, what the High-and Middle-creativity-quality people did are in the same category (both of them had an association process) while the creativity quality levels are different.

However, it is difficult to define the boundary between common association and remote association. Considering the fact that both common association and remote association were reported by participants, the reported common associations and remote associations may not be reliable because participants cannot distinguish between the two. The unreliability may also come from different understandings of remote association and common association. This explanation has further support. In this study, P69 (a High-creativity-quality person) thought of the association of fish with the ocean as a common association, while P33 (a Middle-creativity-quality person) considered the association of fish with the ocean as a remote association.

**Evaluation of the association results.** Nineteen participants mentioned that they evaluated their association results or that they had an evaluation process during their association processes. Most of the participants (20) mentioned that they evaluated their association results. They mainly evaluated whether the association processes were reasonable, whether the association directions were suitable, and whether the results were effective. When they thought that their results were effective, or they felt that they could not be bothered to evaluate the results further, they moved to another cognitive factor process. Seven participants mentioned that they did not evaluate their association results, and most of these individuals (five) were High- and Middle-creativity-quality people, while the remaining two were Low-creativity-quality people. This may be because so few Low-creativity-quality people (18) reported that they engaged in association processes.

**Confidence in the association results.** Sixteen participants reported that they were confident about what they would associate before associating it, while forty-three participants mentioned that they were uncertain. The reason why High-creativity-quality people were uncertain about the results is that the association process is a divergent thinking process for them and what was associated cannot be controlled. For Middle- and Low-creativity-quality people, uncertainty relates to whether their association results will be beneficial to design. Therefore, although High-, Middle-, and Low-creativity-quality people may be uncertain about their association results, the uncertainty areas are different.

**How participants realized that they are using an association process.** There are two ways for participants to realize association processes: when they evaluate the association results, or when they realized that they cannot focus on the design task and that some information unrelated to the design task is generated. Among the participants, 29 reported how they realized their association processes. This number is considerably smaller than that of the participants who realized they had recall processes (N = 49). Most of the High-creativity-quality people (11/14) can realize that they experienced association processes, while only a few of the Middle-creativity-quality people (17/29) and some of the Low-creativity-quality people (8/28) did. This may be because High-creativity-quality people were more familiar with the cognition processes in design. Therefore, they know what they need to do consciously.

**Differences between association and recall.** Four participants reported that recall and association were the same. Fifty-one participants reported that they thought the two were different. Three differences were promoted by the participants: (i) participants can prompt more ideas in association processes than in recall processes (P24); (ii) in recall processes, participants recall fish (the design topic), while in association processes, participants relied on what they recalled to associate more ideas (P64); (iii) association processes generated broad frames of an idea, while recall processes were used to fill in the details of the ideas (P39). Some participants even asserted that the recall process was fundamental to the association process (P62, P18, and P21).

#### 5.1.3. Performance of Combination

Combination is a cognitive process where two or more concepts are mentally synthesized into a new concept. When participants try to combine some information, graphics, or sentences to generate concepts that are incompatible and empty in life, remote combination happened. When participants try to combine some information, graphics, or sentences to generate concepts which are not incompatible in life, common combination happened (Wan and Chiu 2002). Combination processes are often based on recall and association results. Therefore, logically, combination processes often happen after recall and association processes. Sixty-nine participants reported that they have combination processes during the creative design task.

**What the participants combined.** In the combination processes of all three levels of people in this study, participants typically blended fish (the design topic) and products, after which they considered how to make fish and products combine suitably to generate a final product (95% CI = 0.7906~0.9443). The information on fish (the design topic) and products was collected from the recall and association processes. The differences concern the content that was combined. Namely, High-creativity-quality people are more likely to combine a component of a fish with a product (95% CI = 0.4497~0.8866), while Middle- and Low-creativity-quality people are more likely to combine a fish pattern with a product (95% CI = 0.6093~0.8345). This difference revealed why High-creativity-quality people, who recalled a broader array of information on fish and products, may have higher creativity quality levels.

Participants further identified their combinations as common combinations (39), random combinations (17), remote combinations (11), or no combinations (3). There are two ways for participants to justify the category of their combinations. The first method is to judge as to whether they have seen a similar product before. The second method is to judge the relationships between concepts, which often happened in remote or random combinations; however, no matter which method is used, similar to association, participants may have different understanding of different combinations. For example, P12 (a Low-creativity-quality person) mentioned that a combination of a vase and fish was a remote combination, while P7 (a Middle-creativity-quality person) thought that it was a common combination.

**Confidence in the combination results.** Nearly half of the participants (34) had confidence in their results, while a slight majority (seven) were uncertain. High-creativity-quality people were more likely to be uncertain. This may be because they did not limit which kind of products they wanted to design and that they could not know which kind of results they would achieve until the product was finally designed. The reason why participants were certain about their combination results may be because they could predict which kind of product they wanted to design before their combination processes. For these participants, the combination process was simply the act of putting together the information that they obtained from their recall and association processes.

### 5.2. Principal Findings

Our findings suggest that, in terms of recalling, High-creativity-quality people tend to recall a broad topic-related area and products that they feel would have the potential to suit the design topic; Middle-creativity-quality people tend to recall deep information on a specific topic-related area and a specific kind of product. Conversely, Low-creativity-quality people tend to recall the characteristics of the topic-related area, intuitive idea-related information, and some topic-related products. In terms of association, High-creativity-quality people tend to use a few items to generate more related ideas and memories; Middle-creativity-quality people tend to use one item to generate more related ideas and memories. In terms of combination, High-creativity-quality people tend to consider how to suitably combine topic-related information and target products; Middle-creativity-quality people tend to consider how to suitably blend topic-related information and products. Conversely, Low-creativity-quality people tend to combine topic-related patterns and products.

### 5.3. Limitations and Future Research

This study identified the performances of three cognitive factors in creative design and how their performances will affect creativity quality levels. The findings have been discussed; this section will discuss the limitations of this study.

The study focused on the divergent thinking processes of design; however, design processes include divergent thinking and convergent thinking processes. The effects of convergent thinking processes were ignored. Convergent thinking processes were mainly related to participants’ recognition and selection ability. Therefore, participants were asked to think of one design idea with which to mitigate the differences in participants’ recognition and selection abilities. Additionally, participants recruited in this study were from the same background and had similar educational knowledge in design and creativity. The different recognition and selection abilities can thus be controlled.

This study focused on three cognitive factors (recall, association, and combination) that were promoted by existing cognitive processes’ models of creativity. The other cognitive factors, such as working memory, cognitive style, and cognitive load, which can also affect levels of creativity quality, are not detected because these cognitive factors are hard to identify with qualitative methodologies, such as a direct interview. In addition, the effect of working memory, cognitive style, and cognitive load can be reported from the performances of the detected cognitive factors (recall, association, and combination). In other words, working memory, cognitive style, and cognitive load may also affect the three detected cognitive factors. Therefore, although the study did not detect other cognitive factors, such as working memory, cognitive style, and cognitive load, effects of these cognitive factors reflected the detected cognitive factors, which can mitigate the limitations. Considering the fact that this study only reported the performances of cognitive factors that happened in conscious processes, and that unconscious processes cannot be reported, in the future the study will apply more advanced methods, such as eye-tracking and neuroscience technologies, to report the working memory, cognitive style, and cognitive load conditions to detect how these unconscious cognitive factors affected the creativity quality levels. In addition, this study mainly focused on the performance of the contextual details of cognitive factors. The effects of occurrence duration on creativity quality levels were ignored, which are worth detecting in the future.

The creative outputs were assessed by expert raters because experts can assess creativity efficiently and effectively (Amabile 2018; Han et al. 2019; Kaufman et al. 2008; Runco 1994); however, the assessment process is subjective (Zhai et al. 2009). Raters’ experience and knowledge affected the assessment process, and thus the raters themselves may have imposed barriers.

Another limitation concerns the use of the CPSS, as it may not be the most suitable method for assessing creativity. Bias in the assessment results could affect the divisions of the levels of creativity quality. In the future, other creativity assessment methods (such as the consensual assessment technique (CAT; Amabile 1982), the product creativity measurement instrument (PCMI; Horn and Salvendy 2009), or the creative solution diagnosis scale (CSDS; Cropley and Cropley 2008)) may be applied to identify how the different creativity assessment methods may affect the divisions of creativity quality levels.

Furthermore, the creativity quality levels were divided into five levels based on a normal distribution, but it is not certain as to whether this was a good categorization method. Additionally, the study regrouped the five levels into three levels based on the number of participants in each group. This was also a subjective regrouping method, which may also make the division of creativity quality levels unreliable. In the future, the study can further detect peaks, such as why there are more people than others.

Participants in this study were all Chinese people with design backgrounds. Whether the results can be applied to general conditions requires further investigation. Future studies should include larger sample populations.

Considering the fact that cognitive factors were unfamiliar areas for participants whose background had not considered this, this study used a semi-open-choice interview to collect cognitive factors’ performance data; however, the semi-structured interview was conducted after the design task. People may not be able to recall and explain their cognitive performances accurately. Additionally, the semi-open choices may make participants overlook or omit thinking about the real conditions. Future research should therefore collect participants’ cognitive performance during the design process instead of after it. Considering the fact that the semi-structured interview may interrupt the creative design flow if applied in the creative design process, a real-time measurement, such as think-aloud, should be applied. In addition, the interview reported the relationships between the performances of cognitive factors and creativity quality levels in a qualitative way. In the future, some quantitative methods were also expected, such as measuring participants’ memory, association, and combination abilities, in addition to linking these quantitative results with creativity quality levels.

The results were analyzed based on a thematic analysis, where descriptions with similar meanings were grouped. The themes coded in this study, therefore, were affected by the researchers who coded them. In the future, more researchers could be involved in regrouping the themes again to identify whether the coding results are consistent among different researchers.

The results are based on the task to “design a product using the provocation of the word ‘Fish’ within one hour”; however, this is not the only kind of design task. Whether the results can also be applied to other design task forms (such as “using a design to solve a particular problem”) is not certain. In addition, creative design tasks include broader areas, such as services, technologies, and tools, for processes. These areas are also not detected. Furthermore, this study enlarged the findings by using “design topic” to replace the provocation word “Fish”; however, whether the results can be extended requires further discussion. More studies on different design tasks and different design task forms are expected. In addition, the design task was conducted online. The environment of finishing this design task was not controlled, and participants may not have been in a natural designing environment. Therefore, future studies need to be conducted in natural settings, such as design classrooms, and in this way avoid the effect of the environment and make the data more accurate.

## 6. Conclusions

This study aimed to identify the performances of cognitive factors (recall, association, and combination) in creative processes and detect how different cognitive factor performances will affect creativity quality levels. To address these aims, 71 participants were recruited to undertake a creative design task, followed by a semi-structured interview. Five experts in design were recruited to assess the creativity of the design outputs.

The results revealed the relationships between contextual details of cognitive factors and creativity quality levels. For people whose outputs were considered to be of High creativity quality, in the recall process they were more likely to recall a broad topic-related area and products that they felt would have the potential to suit the design topic. Their association processes were mainly based on a few items with which to generate more related ideas and memories. In the combination process, they mainly considered how to suitably combine topic-related information and target products.

For people whose outputs were considered to be of Middle creativity quality, in the recall process they mainly recalled deep information on a specific topic-related area and a specific kind of product. Their association processes were primarily based on one item with which to generate more related ideas and memories. In the combination process, most of them considered how to suitably blend topic-related information and products.

For people whose outputs were considered to be of Low creativity quality, in the recall process they mainly recalled the characteristics of the topic-related area, intuitive idea-related information, and some topic-related products. Few participants in this group mentioned that they had association processes. For these people, their association processes were mainly based on one item with which to generate more related ideas and memories. In the combination process, they mainly combined topic-related patterns with products.

From the results, this study can help explain why people experiencing the same cognitive processes or the same cognitive factors in a creative process may have different creativity quality levels. In addition, the study compared the different performances among High-, Middle-, and Low-creativity-quality-level people. This will be helpful for designers and researchers to understand what curbs the development of creativity. This study also brings clues for deeply understanding cognitive processes in creativity through understanding the performances of cognitive factors. The findings of this study can be used to help researchers and designers better understand the performances of creativity-related cognitive factors and how they are related to creativity quality levels. By understanding the relationships, this study has the potential to help designers to introspect on their performances of cognitive factors in a creative design and self-assess as to whether their outcomes are creative or not. By following the performances associated with creative outputs’ quality levels, designers’ abilities to generate creative ideas may also increase.

## Figures and Tables

**Table 1:** Cognitive factors that are involved in the reviewed cognitive process models.

Cognitive Process Model	Cognitive Factors Included in Divergent Thinking Cognitive Processes
Blind variation and selective retention model (BVSR)	Memory, combination
Genoplore model	Long-term memory, association, and combination
Gabora’s model	Related association, remote association
Structure of intellect model	Association, combination
Cognitive factor process model	Long-term memory, short-term memory
Nijstad et al.’s model	Remote association

**Table 2:** Eighteen criteria of the CPSS in this paper (adapted from Chulvi et al. (2012), Han (2018), and Yin et al. (2021)).

Subscales	Items	
Novelty	Average–revolutionary	Common–astounding
	Commonplace–astonishing	Commonplace–original
	Customary–surprising	Ineffective–effective
	Overused–fresh	Predictable–novel
	Usual–unusual	
Usefulness	Inappropriate–appropriate	Inoperable–operable
	Nonfunctional–functional	Inadequate–adequate
	Unnecessary–necessary	Inessential–essential
	Unusable–usable	Unfeasible–feasible
	Useless–useful	

**Table 3:** Descriptive statistics (minimum, maximum, mean, and SD value) of each expert’s CPSS results. (The CPSS score ranges from 0 to 126).

Data Source	Experts’ Results	Descriptive Statistics			
		Minimum	Maximum	Mean	Std. Deviation
Based on original data	Expert 1_CPSS	20.0	126.0	72.585	32.9304
	Expert 2_CPSS	27.0	115.0	73.972	22.1809
	Expert 3_CPSS	30.0	103.0	70.408	18.9807
	Expert 4_CPSS	43.0	119.0	87.070	21.0625
	Expert 5_CPSS	43.0	108.0	80.831	15.5423
Based on normalized data	Expert 1_CPSS	0.00	1.00	0.4868	0.30696
	Expert 2_CPSS	0.10	1.00	0.5423	0.21897
	Expert 3_CPSS	0.00	0.93	0.5446	0.24483
	Expert 4_CPSS	0.01	1.00	0.6104	0.27252
	Expert 5_CPSS	0.00	1.00	0.6059	0.20022

**Table 4:** The division index for creativity quality levels.

Creativity Quality Level		μ = 0, σ = 1	Real Percentage	Simplify Percentage	Percentage Range	Score (If With Infinity Samples)	Sample Number from This Study
High	Outstanding	>μ + 1.5σ	6.55%	5%	Top 5%	0.95–1	0
	Excellent	(μ + 0.5σ)~(μ + 1.5σ)	30.85%	30%	Top 5–30%	0.7–0.95	14
Middle	Very good	μ~(μ + 0.5σ)	50%	50%	Top 30–50%	0.5–0.7	29
Low	Satisfactory	μ~(0.5σ − μ)	69.15%	70%	Top 50–70%	0.3–0.5	21
	Not so good	<μ − 0.5σ	100%	100%	Top 70–100%	0–0.3	7

**Table 5:** Transformation from interview questions to themes.

Initial Themes	Interview Questions	Transformed Codes
Recall	What is the aim of recall?	Aim of recall
	How much confidence do you have in the recall results before having recall processes?	Confidence in recall
	Can you provide more information on what you have recalled during this recall process?	Contextual details
	Which is the display form of the recall process in your mind?	Display form
	How do you know you need a recall process?	How to realize the need for recall
	How do you realize you need a recall process?	How to realize recall
	Do you think you use the results from recall quickly or hard?	Working memory
	Do you meet any difficulties in the recall process?	Difficulty
	Do you evaluate the results you have searched in the recall process?	Evaluation
	What is the difference between association and recall processes of you?	Relations between association and recall
Association	What is the form of association?	Association form
	How much confidence do you have in the association results before having association processes?	Confidence in association
	Can you provide more information on what you have associated during this association process?	Contextual details
	Which is the display form of the association process in your mind?	Display form
	How do you know you need an association process?	How to realize the need of association
	How do you realize you need an association process?	How to realize association
	Do you think you use the results from association quickly or hard?	Working memory
	Do you meet any difficulties in the association process?	Difficulty
Combination	What is the difference between association and combination processes of you?	Combination and association
	Which is the display form of the combination process in your mind?	Display form
	Are the combination results consistent with your expectation?	Whether the same with expectation
	How much confidence do you have in the combination results before having combination processes?	Confidence in combination
	Can you provide more information on what you have combined during this combination process?	Contextual details
	How do you know you need a combination process?	How to realize the need of combination
	How do you realize you need a combination process?	How to realize combination
	Do you think you use the results from combination quickly or hard?	Working memory
	Do you meet any difficulties in the combination process?	Difficulty

## Data Availability

The data presented in this study are available in the article.
